# Preliminary Evidence for Feasibility, Use, and Acceptability of Individualized Texting for Adherence Building for Antiretroviral Adherence and Substance Use Assessment among HIV-Infected Methamphetamine Users

**DOI:** 10.1155/2013/585143

**Published:** 2013-09-03

**Authors:** David J. Moore, Jessica L. Montoya, Kaitlin Blackstone, Alexandra Rooney, Ben Gouaux, Shereen Georges, Colin A. Depp, J. Hampton Atkinson, The TMARC Group

**Affiliations:** ^1^Department of Psychiatry, School of Medicine, University of California, San Diego, La Jolla, CA 92037, USA; ^2^HIV Neurobehavioral Research Program, 220 Dickinson Street, Suite B (8231), San Diego, CA 92103, USA; ^3^San Diego State University/University of California, San Diego Joint Doctoral Program in Clinical Psychology, San Diego, CA 92120, USA; ^4^VA, San Diego Healthcare System, San Diego, CA 92161, USA

## Abstract

The feasibility, use, and acceptability of text messages to track methamphetamine use and promote antiretroviral treatment (ART) adherence among HIV-infected methamphetamine users was examined. From an ongoing randomized controlled trial, 30-day text response rates of participants assigned to the intervention (individualized texting for adherence building (iTAB), *n* = 20) were compared to those in the active comparison condition (*n* = 9). Both groups received daily texts assessing methamphetamine use, and the iTAB group additionally received personalized daily ART adherence reminder texts. Response rate for methamphetamine use texts was 72.9% with methamphetamine use endorsed 14.7% of the time. Text-derived methamphetamine use data was correlated with data from a structured substance use interview covering the same time period (*P* < 0.05). The iTAB group responded to 69.0% of adherence reminder texts; among those responses, 81.8% endorsed taking ART medication. Standardized feedback questionnaire responses indicated little difficulty with the texts, satisfaction with the study, and beliefs that future text-based interventions would be helpful. Moreover, most participants believed the intervention reduced methamphetamine use and improved adherence. Qualitative feedback regarding the intervention was positive. Future studies will refine and improve iTAB for optimal acceptability and efficacy. This trial is registered with ClinicalTrials.gov NCT01317277.

## 1. Introduction

Mobile health (mHealth) interventions aiming to enhance health behaviors have recently proliferated [1]. mHealth strategies are designed to be integrated into the everyday lives of patients in order to minimize barriers to intervention implementation and facilitate use and generalizability [2]. Both the mobility and popularity of cell phones make it possible to remotely deliver services to assist people with behavior modification and disease self-management [3], thereby improving health outcomes. Short-message service (SMS; i.e., text messaging), in particular, represents a low-cost route to promoting health behaviors, such as treatment adherence, due to the ubiquitous nature of this technology on mobile devices. Furthermore, SMS technology supports interactivity (e.g., two-way communication) and can be personalized at the individual level [4, 5].

Thoughtful mHealth interventions grounded in behavior change theory may therefore be particularly advantageous in advancing aspects of health care (e.g., delivery and assessment). Despite relatively few high-quality randomized controlled trials (RCTs) supporting mHealth tools, several interventions to improve adherence to antiretroviral therapy (ART) among persons infected with human immunodeficiency virus (HIV) have yielded positive results [1]. ART is currently the standard of care for persons infected with HIV, and effective adherence to ART is the key to deriving therapeutic benefit [6, 7]. SMS-based interventions have begun to show efficacy in promoting ART adherence in RCTs but are currently in the early stages of development and refinement [8–10]. mHealth interventions have potential to decrease barriers to traditional ART adherence interventions, particularly in difficult-to-track groups because they ameliorate obstacles such as transportation, insurance, and physical limitations [11]. Substance users are one such high-risk subgroup of persons living with HIV who have been documented to be especially nonadherent to ART [12, 13]. Taken together, HIV+ substance users may represent both a critical and feasible target of public health significance for such mHealth adherence interventions.

In addition to ART adherence, substance use behaviors may also be a potential target for assessment or modification via mHealth interventions. Notably, a recent survey involving patients in substance abuse treatment documented that the vast majority of patients reported having access to mobile phones (91%) and to text messaging (79%) [14]. Challenges remain in accurately assessing risk of relapse among substance users, and mHealth technologies may be able to assist by obtaining “real time” data, such as self-reported mood and engagement in substance use. This “real time” data could potentially enable earlier relapse intervention and/or keep individuals continuously engaged in treatment. The deployment of mHealth for substance use disorder treatments is a developing area of research, and early work in this field is promising (see [15] for a review). To our knowledge, investigations using mHealth to promote ART adherence have not yet included or targeted persons with active substance use [16]. The use of mHealth technologies may therefore be efficacious in simultaneously monitoring and assessing medication adherence and substance use among persons with HIV infection and co-occurring substance use problems.

In the context of HIV infection, methamphetamine use may be a particularly relevant substance of abuse given the high comorbidity rate between these two conditions [17]. In fact, methamphetamine users are more likely to be HIV infected than opioid users in the western United States [18], which is primarily facilitated by the link between methamphetamine use and risky sexual behaviors [19]. Importantly, recent methamphetamine use is particularly predictive of poor ART adherence (e.g., [13, 20–22]). Given that suboptimal ART adherence can lead to virologic rebound, development of medication-resistant strains of HIV, and more rapid progression to AIDS and death [23–25], sustained treatment and ART adherence is critical. 

Based on the growing evidence supporting mHealth assessment and intervention, the ubiquity of SMS technology, and the critical need to improve ART adherence among persons with methamphetamine use and HIV infection, the overarching goal of the parent study was to develop and evaluate an SMS intervention to improve ART medication adherence among persons with methamphetamine use and HIV infection. While the RCT of the developed intervention (individualized texting for adherence building (iTAB)) is ongoing, the goals of this present study were to use preliminary data to (1) examine response rates to text messages regarding methamphetamine use and medication adherence, (2) determine whether our assessment of methamphetamine use gathered via SMS is consistent with self-report information of substance use gathered in a clinical interview (i.e., construct validity for methamphetamine use assessment via SMS), and (3) summarize preliminary participant feedback of the ongoing intervention. The rationale for reporting these preliminary data is driven by recent publications suggesting a more rapid approach to publishing behavioral intervention data, especially as it relates to the rapid dissemination of the content of mHealth interventions (e.g., [26–28]). The information presented herein may be informative for the development of other mHealth interventions to improve health outcomes in difficult-to-treat individuals. 

## 2. Method

### 2.1. Participants

This report represents results from the first 29 HIV-infected active methamphetamine-dependent individuals (i.e., use within 30 days of baseline) enrolled in an ongoing pilot RCT designed to improve or maintain ART medication adherence. Target enrollment for this ongoing study is 50 individuals in the active condition (iTAB) and 25 individuals in the active comparison condition. The unbalanced design was chosen to maximize the ability to investigate the data from within the iTAB group. Of the 29 individuals presented here, 20 were assigned to the iTAB arm and 9 were assigned to the control arm. As this study was still ongoing, data were not available for all subjects for all outcomes. Analyses of SMS and substance use data included 21 participants (13 iTAB, 8 control); analyses of feedback questionnaires included 26 participants (17 iTAB, 9 control); and analyses of qualitative feedback interviews included 19 participants (12 iTAB, 7 control). The UCSD Human Research Protection Program approved the current study. Participants provided written informed consent to participate.

Inclusion criteria were the capacity to provide informed consent, age 18 years or older at enrollment, documentation of HIV infection, self-reported methamphetamine use within the last 30 days, DSM-IV-TR diagnosis of methamphetamine abuse or dependence via the Composite International Diagnostic Interview [29], and an active prescription for an antiretroviral medication. Participants also had to be willing to respond to text messages and utilize electronic medication tracking devices (i.e., medication event monitoring system as previously described [30]) for the identified antiretroviral medication over the study period. Participants needed to show capability of responding to text messages at baseline by direct observation. Exclusion criteria were minimal in order to enhance generalizability and recruitment feasibility in that many of these individuals had several co-occurring conditions (e.g., psychiatric disorders, hepatitis C virus). Of note, plasma HIV viral load detectability was not an inclusionary criterion for the present study. Given that (1) methamphetamine use is a well-established risk factor for antiretroviral nonadherence, (2) self-reported antiretroviral adherence tends to overestimate actual adherence, (3) viral load detectability is dynamic, and costly to gather at a screening visit, and (4) recruitment of actively using methamphetamine HIV+ persons is difficult, we chose to enroll persons with both detectable and undetectable HIV viral loads.

Participants received monetary incentives for both the initial ($50) and follow-up assessments ($60). Participants were encouraged to use their own cell phones and were reimbursed for any additional costs incurred by participating in the study over their regular cell phone use. A mobile phone, not a smartphone, with a comprehensive texting plan was loaned to those participants who did not own a cell phone or were unable to receive text messages on their current phone (ten of 29 participants were provided a cell phone for use on the study).

### 2.2. Focus Groups and Intervention Development

The intervention was developed by means of a user-centered approach. Two focus groups, each with ten persons with methamphetamine abuse or dependence and HIV infection (not enrolled in the current study), were conducted to assess the feasibility of a text message intervention to improve adherence among this population, as well as to aid the development of SMS content for the intervention. In brief, focus group participants were recruited from large ongoing research studies of HIV infection and substance use. The focus groups generated broad barriers and facilitators for adherence and preferences for personalized reminder text messages to promote adherence using an mHealth intervention. Findings from these focus groups are described in a separate manuscript [31]. As a result of these focus groups, 40 reminder text messages that fall into eight reminder themes were developed for use in the intervention. We piloted the intervention with five individuals (data not included in the current study) after the initial development and made further minor modifications accordingly.

### 2.3. Pilot Randomized Controlled Trial

These interim results represent randomized participants with the outcomes of interest (i.e., this was not an intent-to-treat analysis). Both the iTAB (*n* = 20) and control groups (*n* = 9) received the following intervention components. 

#### 2.3.1. Medication Adherence Education

The medication adherence education included multiple components of previously successful medication interventions and published barriers to successful medication adherence among substance users [12, 13, 32, 33]. The adherence education presented the importance of attention to medication maintenance, health benefits of adherence to ART medication, adverse medication and methamphetamine use effects, problems of adherence for methamphetamine users, and practical medication adherence strategies. The medication adherence psychoeducation was delivered via PowerPoint, lasted approximately 30 minutes, and provided time for the participants to ask questions and speak about their own experiences adhering to medications. 

#### 2.3.2. Creation Process of Personalized Reminder and Reinforcement Text Messages

During the medication adherence presentation, all participants were informed about the use of reminder strategies (e.g., creating a “note to self” to put in a visible place or writing a reminder on a calendar) to facilitate their antiretroviral adherence. Participants assigned to iTAB then selected, modified, and/or created ten personalized reminder text messages working from a list of 40 predetermined text message reminders. Participants in the control group also selected ten messages from the same list that were printed on one sheet of plain white paper for them to take home and use as they desired. For example, a participant might write the messages on sticky notes around his or her home or set reminders on their own phones as discussed in the psychoeducational portion of the study. The control group did not receive daily ART reminder text messages during the intervention. 

In addition to the personalized reminder text messages, participants in the iTAB group also selected ten reinforcement text messages working from a list of 20 predetermined choices (e.g., “Great job, every dose helps” and “Keep up the good work.”). Participants also had the option of writing their own reinforcement text messages and/or modifying the existing messages. The reinforcement text messages were sent to reinforce events where the participant reported taking his or her medication.

#### 2.3.3. Text Messages to Evaluate Daily Methamphetamine Use

Both groups received a daily text message asking if they had used methamphetamine in the last 24 hours. To protect the participants from any potential legal or personal ramifications associated with disclosure of methamphetamine use, the word “methamphetamine,” or variants thereof, were not included in the text messages. Instead, as a proxy for a direct question about methamphetamine use, at the baseline visit, participants were instructed to respond to a daily 9 a.m. message inquiring: “Have you done anything in the past 24 hours? (Y) yes (N) no.” It was further emphasized that answering either “yes” or “no” to this question would not impact individuals' participation in the adherence study. 

### 2.4. iTAB Specific Intervention Components

In addition to selecting individualized reminder and reinforcement text messages, participants in the iTAB group provided his/her preferred name and a description of their tracked medication (e.g., “the white pill”) to be used in the messages. Participants were guided to use a description of the medication rather than the name of the medication itself in order to avoid a potentially stigmatizing medication name appearing in the content of the text message. The participant and examiner identified appropriate time(s) for the reminder text message (i.e., once daily or twice daily, depending on the instructions for the ART regimen). An example reminder message might read, “John, it's med time! Pls take ur big blue pill now. Pls reply (A) took (D) didn't (G) snooze.” A reinforcement message might read, “Great job! Ur current adherence: 75%. Adhr when u take ur next dose: 80% (4/5 doses).”

Additionally, the automated system sent out a “noncompliance” message to the participant after three consecutive days of missed messages, and an alert was sent to the study coordinator. The study coordinator had real-time access to participant response logs to identify problems and contact participants who were having difficulties responding to the system (i.e., two days after “noncompliance” message if still no response).

### 2.5. Intervention Feedback

At the final visit, participants were given a standardized feedback questionnaire using Likert-type response options. Questions addressed ease of understanding/problems with reminder text messages, overall satisfaction with the study, self-perceived efficacy as it relates to participation in the study, and likelihood of using the system in the future. Questions with response options are listed in Table 2. To bolster the feedback questionnaire data, participants completed a semistructured feedback interview regarding their involvement in the study. Specifically, participants were asked to describe their experience in participating in the study and to comment on the text messages. 

### 2.6. Other Assessments

#### 2.6.1. 30-Day Substance Use Interview

At followup, subjects were administered a detailed substance use interview, recording both frequency and quantity of methamphetamine use. To allow for direct comparison to text message responses regarding methamphetamine use, only methamphetamine use during the 30-day study period was analyzed. Similarly, only the last 30 days of text message data were considered for subjects whose visit interval covered a period longer than 30 days. 

### 2.7. Statistical Analyses

Comparison of positive versus negative responses to the SMS methamphetamine use messages (i.e., use versus nonuse) was examined using a matchedpairs *t*-test. Additionally, analyses examining associations between SMS methamphetamine use responses and self-reported substance use obtained by the 30-day Substance Use Interview were determined using nonparametric Spearman's rho correlations. The standardized feedback questionnaire data were summarized as response proportions for various Likert-type scales. Pearson chi-squared tests were conducted to compare responses on the standardized feedback questionnaire. Quantitative statistical analyses were performed using JMP 9.0.2 Statistical Software.

Transcripts of the semistructured feedback interview were analyzed in the following manner. The content of each interview was audio taped and subsequently transcribed by a single study investigator (Shereen Georges). The transcripts were then independently coded, based on emergent themes, by two investigators (Jessica L. Montoya & Shereen Georges). Segments of the transcript could be assigned more than one code. Disagreements in description or assignment of codes were resolved by consensus among investigators and led to the refinement of codes. The final coding structure of the transcripts was reviewed to determine the level of agreement in the codes applied. Data analysis was performed using QSR International's NVivo9 qualitative data analysis software.

## 3. Results

### 3.1. Demographics and Sample Characteristics

Participants in the present study were, on average, middle-aged non-Caucasian males with approximately one year of college education. In terms of HIV disease, approximately two-thirds had undetectable viral loads. Details of the sample are provided in Table 1. There were no significant differences between the groups for any of the variables shown. Participants were monitored for an average of 29.9 days (range: 29-30).

### 3.2. iTAB Condition: ART Reminder Text Messages

**Figure 1:**
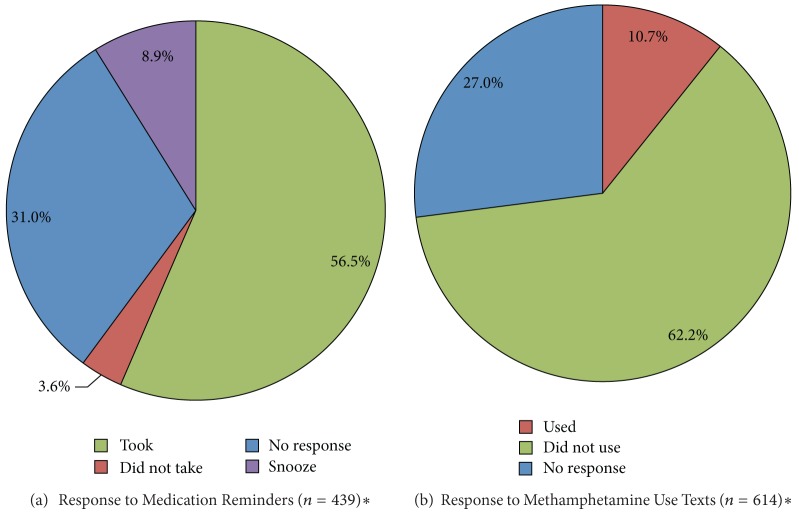
Response patterns for (a) medication adherence reminder text messages and (b) methamphetamine-use text messages. Note: ∗ Sample size represents number of messages sent to participants not the number of participants on study.

Among persons assigned to the iTAB condition, the overall mean response rate to medication reminder text messages was 69.0%. Participants rarely responded that they did not take ART medications (3.6%).  Figure 1(a) shows the response pattern to the adherence reminder text messages. 

Using a matched pairs analysis among the iTAB group, participants were significantly more likely to respond that they had been adherent than to indicate nonadherence (“took” responses: *M* = 19.08, SD = 9.3 versus “didn't take” responses: *M* = 1.23, SD = 2.1; *t*  (df = 12) = −6.52, *P* < 0.001).

Among the received responses to adherence messages, we examined the proportion of responses indicating that the individual took his/her medications (81.8%), did not take his/her medication (5.3%), or sent a snooze response indicating that they would like to receive a reminder in an hour (12.9%). That is, the denominator used in these calculations represents the number of received participant responses of any type (i.e., “took,” “did not,” or “snooze”), but does not include instances where the participant failed to respond to the adherence text message.

### 3.3. Text Message Assessment of Methamphetamine Use

The overall mean response rate to the methamphetamine use text messages was 72.9% (*M* = 21.3 responses per participant), while the overall mean nonresponse rate was 27.0% (*M* = 7.9 nonresponses per participant). 

Examining response patterns among participants in both groups, we observed that participants were more likely to indicate that they were not using methamphetamine via the SMS messages than to indicate that they were using methamphetamine (“no”: 18.2 days, 62.2% versus “yes”: 3.1 days, 10.7%; *t* = 10.3  (df = 20), *P* < 0.001). An overall pie chart showing response rates for the SMS methamphetamine question, including instances where the participant did not respond, is shown in Figure 1(b).

Similar to the approach used above for the adherence messages and in order to control for instances where participants failed to respond to SMS message of methamphetamine use, relative values of methamphetamine use and abstinence were calculated by dividing the number of SMS messages indicating use or nonuse by the number of total responses by the participant (versus across the total study period). Using this method, participants indicated adjusted methamphetamine use 14.7% of the time and non-use 85.3% of the time across the study period.

### 3.4. Comparison of 30-Day Substance Use Interview and Daily Methamphetamine Text Message Data

Using data derived from the examiner administered semistructured interview reviewing methamphetamine use over the 30-day study period (and thus directly overlapping with the time period of SMS methamphetamine use reporting), participants reported actively using methamphetamine 27.3% of the time (*M* = 8.2 days). The number of SMS messages endorsing methamphetamine use was significantly correlated with the number of self-reported days of active methamphetamine use over the study period on the substance use interview (*ρ* = 0.65, *P* = 0.001). Importantly, the number of SMS messages denying methamphetamine use was not associated with days of methamphetamine use on the substance use interview (*ρ* = −0.17, *P* = 0.46), indicating divergent validity supporting SMS assessment of methamphetamine use. Non-response to SMS messages was not associated with number of days of methamphetamine use as reported during the interview (*ρ* = −0.14, *P* = 0.54).

### 3.5. iTAB Standardized Questionnaire Feedback 

There were no statistically significant group differences on the standardized feedback questionnaire (*P* > 0.05; see Table 2). In terms of feedback on the text messages, participants across both groups reported no difficulties with understanding the text messages (94% iTAB versus 89% control). Additionally, the majority of participants indicated that they experienced no interference with their daily activities by receiving daily text messages (76% iTAB versus 100% control). Similarly, results indicated high overall satisfaction with participation in this study (65% iTAB versus 44% control reported being “extremely satisfied”). In terms of self-perceived efficacy, iTAB participants reported that the daily methamphetamine text message (i.e., “Have you done anything in the past 24 hours?”) may have influenced their use behaviors: 35% reported they used “a lot less,” 35% reported they used “a little less,” and 24% reported using “about the same.” Control participants, on the other hand, reported using methamphetamine “about the same” 44% of the time, while 22% of controls reported using “a lot less” and 33% reported using “a little less.” Responses related to intervention influences on changes in ART medication adherence were as follows: “about the same” (29% iTAB versus 33% control), “a little better” (24% iTAB versus 56% control), and “much better” (35% iTAB versus 0% control). Overall, most participants indicated that they would participate in similar studies in the future (71% iTAB versus 78% control) and that a text messaging intervention could be “very much” helpful to them in the future (59% iTAB versus 44% control).

### 3.6. Qualitative Feedback of Intervention

Analysis of the semistructured feedback interviews demonstrated high degree of concordance between raters of nine identified themes of 171 coded statements (mean *κ* = 93.3, SD = 0.21). When prompted to describe their participation experience, 17 persons indicated experiences that were coded as “positive.” The following participant quotation provides an example of a positive experience response.

*“It [the study] was interesting. The reminders were helpful as far as reminding me to take my meds […] The text asking if I've done anything in the past 24 hours was helpful because it actually made me ask myself on a daily basis if I did anything.”*   (iTAB 1)**

Another participant expressed enthusiasm about the study, as it related to the daily text messages and the supportive nature of the text message content.

*“I loved the day-to-day messages that I got. It was reassuring and comforting, and it was just nice knowing someone was out there looking after me.”*  (iTAB 2)**

Not all respondents, however, indicated positive experiences. Such responses were coded as “negative” experiences in the analyses. Two respondents reported noted not liking certain aspects of the study. For example, one participant indicated a negative experience as it related to the questions about methamphetamine use and how it made him feel about taking medications.

*“The study had a suggestive impact on my behavior which was that, rather than just to monitor my behavior, I experienced much more recreational drug use than I would have participated in had I not been in the study. I used drugs much more frequently and much more than I have ever used drugs; and to a more severe degree […] It sort of, overall, made me resent taking the medications which I haven't felt that before. I've been taking HIV meds for the last 2 years.”*  (iTAB 3)**

In summary, the majority of participants' interview feedback content was coded as indicating a “positive” experience, while a minority experienced “negative” consequences from participation in the study. 

When asked during the feedback interview what they thought about the text messages they received during the study, the participants offered varied free responses. The following seven themes about the text messages were coded from the participants' responses: likeable, helpful, easy, annoying, improved with time, tiring with time, and unlikeable. Many participants (i.e., one control and ten iTAB participants) expressed liking the texts in general. For example, one participant stated

*“I like them [the text messages]. I like how they were all different.”*  (iTAB 4)**

Three participants reported that they found the text messages helpful. For example, one participant stated the following.

*“[The text was] very nice; it was very good. Good reminder. It helped me stay on track.”*  (iTAB 5)**

One control participant indicated that he found using the text messaging system to be easy.

*“I feel like it was easy. Once I developed the position of all the keys, I was able to do it in my sleep almost.”*  (Control 1)**

One control participant described feeling annoyed at times by the text messages.

*“Sometimes they were annoying.”*  (Control 2)**

One participant who indicated initial difficulty with the system indicated that the text messages improved with time.

*“First bad, but then [the text messages] got better. I think I just made it harder on myself […] The first few days were overwhelming, but after you explained it to me, it was fine.”*  (Control 3)**

In contrast, one iTAB participant reported finding the texts to be initially good but then tiring with time.

*“Good, it got a little tiring towards the end.”*  (iTAB 2)**

In summary, participants indicated varied, although mostly positive, thoughts in regard to the text messages. 

## 4. Discussion

The present study shows preliminary evidence of feasibility and acceptability of an SMS intervention to gather data on methamphetamine use and to provide adherence reminders among persons with HIV infection and recent methamphetamine use. The significant positive correlation between SMS and interview-based methamphetamine use reports provides preliminary support for the construct validity of methamphetamine use assessment via SMS. Participants also responded to the adherence reminder message system approximately two-thirds of the time, reported very few difficulties in understanding the messages, and provided positive feedback regarding the intervention. Thus, an SMS messaging system targeting both substance use evaluation and medication adherence improvement appears feasible to implement in this difficult-to-treat group.

Accurately capturing details of substance use has traditionally posed a challenging problem for substance use researchers and providers [34]. There have been recent advances using portable technology for real-time monitoring of drug cravings and use (e.g., [35]). Our preliminary evidence is consistent with these previous publications. Using calculations from the days when participants responded to SMS messages, individuals reported methamphetamine use on approximately 14.7% of days (as compared to 27.3% using a retrospective interview approach). The slightly lower rate of methamphetamine use as obtained via SMS may be attributed to (1) the potential that, on the days that participants failed to respond, they may have been using methamphetamine, (2) a hesitancy from some individuals to report methamphetamine use via SMS, (3) the difficulty of recalling information over longer periods of time for the interview, which was conducted at the final study visit, (4) the use of a nondirect question about methamphetamine use to secure participant privacy, and/or (5) a combination of these factors. It is important to note that the SMS assessment of methamphetamine use was embedded in a study focused on medication adherence and therefore was not the exclusive focus of the study. Moreover, it is possible that receiving text message inquiries about methamphetamine use during the study may have influenced retrospective self-reporting of substance use behavior at the study follow-up visit.

On the structured feedback questionnaire, 70% of persons reported that they believed that the daily methamphetamine use text message made them use a little to a lot less methamphetamine. Qualitative feedback gathered from a semistructured interview supported the idea that, in general, participants viewed the methamphetamine text messages favorably because they helped maintain the goal of abstinence. For example, during the interview one participant described how the methamphetamine text message kept abstinence at the forefront of his mind. Thus, self-monitoring methamphetamine use via text messages may be a useful and an easy way for participants to monitor and/or gain insight to the frequency of their methamphetamine use. This may be particularly important for a group of individuals that are known to have attention and memory deficits [36].

In acknowledgment of the potential detrimental effects of inquiring about methamphetamine use on a daily basis, we observed one participant who believed that the daily messages regarding methamphetamine use may have served as a trigger for subsequent and continued use. On the standardized feedback questionnaire, this same participant endorsed that the daily methamphetamine use text made him use “a lot more.” Additionally, this participant described feeling resentment regarding the need to take medications as a result of his participation in the study, even though he had been on an ART regimen for the prior two years. Although the majority of participants indicated satisfaction with the various study components, a text message specifically inquiring about substance use may not be appropriate for all current substance users. Further research is needed to determine the individual factors that influence positive and negative experiences of a daily assessment of substance use behaviors.

Participants enrolled in the iTAB condition demonstrated similar engagement with the adherence text messages as was illustrated with the methamphetamine messages (i.e., iTAB participants responded to 69.0% of the adherence reminder text messages). One interesting response pattern is that participants rarely chose the “didn't [take]” response to the adherence reminder texts. This response pattern may reflect the possibility that the participants were, in fact, largely ART adherent and simply forgot, or did not have time, to respond to the text message promptly. Alternatively, the results may indicate that participants opted not to respond rather than admit nonadherence. Given that there is rich data in why individuals fail to take medications, subsequent interventions and feedback questions should focus on why participants rarely choose the option of reporting missed doses; future studies could then incorporate this information in development of novel approaches to better ascertain those data (e.g., softer language or reinforcers, such as “did not get to it today,” “you'll get it next time!” versus simply “did not take”). The current iTAB system is designed such that a “didn't [take]” response triggers a follow-up text regarding reasons for the missed dose. Participants may have wanted to avoid this additional text. Responses to the feedback questionnaire item, “Receiving text messages interfered with my daily activities,” provides some indication that fewer messages may have been more optimal. Specifically, none of the control participants indicated that the daily messages interfered with daily activities whereas 24% of the iTAB participants endorsed some interference with daily activities. Of note, iTAB participants received more text messages than the control participants thereby adding to the overall burden of participation for iTAB participants and possibly negatively impacting responding rates. In addition, there is some indication in mHealth HIV adherence research suggesting that fewer text messages may be optimal for adherence and participant engagement and satisfaction [37]. Future research is needed to tackle the trade-off between providing fewer text messages (to improve participant acceptability) and providing a sufficiently intense intervention to be effective (improve adherence). Moreover, the research literature has not yet explored the possibility of allowing participants the ability to control the frequency of messages. Detailed examinations of the content of adherence messages, ranging from simple messages not specifically addressing adherence (e.g., “How are you?”) to more complex messages intended to be motivating and targeted at health promotion (e.g., “People care about you…”, “Not taking your meds could make you resistant…”) are also warranted. Finally, determining whether there are specific HIV-infected subpopulations for which a given type of messaging may or may not work is also worthy of investigation.

Although there were not significant differences between the iTAB and control groups on the standardized feedback questionnaire, participants generally reported a positive experience. Explicitly, the feedback from participants showed that 94% of iTAB individuals were at least somewhat satisfied with the intervention as compared to 66% of control participants, 35% of individuals in the iTAB group reported that the intervention made their medication adherence “much better” as compared to 0% of individuals in the control group endorsing this response, indicating some specificity of the ART text messages to adherence behaviors. Perhaps more interesting is the fact that 56% of the control group felt that the text messages about methamphetamine use made adherence at least “a little better.” Therefore, participants in the control condition may have generalized their engagement with substance use assessment text messages (i.e., methamphetamine use) to other health behaviors beyond the content of the messages (i.e., adherence). Thus, reminder messages, perhaps regardless of content, in the context of a stated goal to improve medication adherence may be useful. The process of receiving messages on a daily basis may, therefore, instill a sense of health behavior accountability in participants. Additionally, data from the open-ended feedback interview suggests the possibility that participants felt supported by the intervention. This finding is consistent with previous work in which social support has been identified as an important factor for positive adherence outcomes [33]. Participants also clearly indicated that they would be willing to participate in future studies, with approximately three-quarters of individuals endorsing that they would “very much” like to participate in future studies of this type. Participants additionally indicated that a text messaging intervention such as the study described here would be “very much” helpful (59%) to them in the future. These data suggest that interventions such as the one described here may be scalable and that uptake may be feasible in future studies.

There are several limitations to the current study that should be mentioned. This was a small sample of convenience taken from an ongoing RCT. As a result, the data are more descriptive than is typically reported, and we do not yet have objective outcome data on whether the intervention changed the target behavior of adherence or non-target behaviors such as substance use. With that said, participants were generally responsive and positive about the intervention. We cannot rule out that any perceived benefits of the study simply represented subject-expectancy effects (e.g., the participant feels compelled to say she or he liked the intervention). An additional limitation of reporting data from an ongoing RCT was that data were not available for all subjects for all outcomes. Recent research advocates the use of imputation-based strategies to handle “nonignorable” missing data [38], which future analyses may employ. As previously noted, a measure of ART adherence was not used as inclusionary/exclusionary criteria. Thus, although the study was designed to improve ART adherence among active methamphetamine users, it is possible that the study features are only capable of maintaining, worsening, or having no effect on adherence for already adherent participants. Finally, we are not able to examine predictors of non-adherence at the present time because outcome data are still pending. Nonetheless, the information provided, specifically as it relates to the feasibility and validity of the SMS methamphetamine use, is novel.

Future directions for mHealth interventions are numerous. Specifically, future mHealth interventions could target the reduction of substance use behaviors by replicating components of traditional substance use interventions in supportive text messaging. Alternatively, interventions could build on existing social networks by texting a friend or family member when substance use is reported, take advantage of geolocation tools by sending messages about areas that may serve as triggers for substance use, and/or provide resource information such time and location of the next Narcotics Anonymous meeting. There are challenges with the larger implementation of these interventions as well, such as who would fund or support the messaging systems in the clinic setting. For optimal delivery, systems would need to be integrated into existing large-scale electronic health systems.

## 5. Conclusions

In summary, the results of this preliminary analysis of an ongoing RCT to improve medication adherence and assess methamphetamine use show that SMS messaging is feasible, acceptable, and perceived to be helpful. Importantly, we provide initial support that endorsement of methamphetamine use via text messaging was externally valid in comparison to retrospective reports. Results regarding the ability of the iTAB intervention to lead to tangible changes in adherence behavior are pending the completion of this trial. mHealth interventions offer opportunities for reaching challenging and marginalized populations and may be a useful and low-cost approach to improving the health of people with co-occurring HIV infection and methamphetamine abuse or dependence.

## Figures and Tables

**Table 1:** Descriptive characteristics of the study groups (*N* = 29).

	iTAB (*n* = 20)	Control (*n* = 9)
Demographics		
Age; mean (SD)	46.8 (8.3)	52.4 (6.6)
Education; mean (SD)	13.2 (2.7)	14.3 (2.7)
Male; % (#)	90.0% (18)	100.0% (9)
Caucasian; % (#)	55.0% (11)	33.3% (3)
HIV disease characteristics		
CD4 count; median [IQR]^a^	586.5 [140.5, 974.8]	606.5 [198.3, 1053.8]
Nadir CD4 count; median [IQR]^b^	148 [14.8, 493.8]	235 [153, 362.5]
HIV RNA plasma; median [IQR]^c^	1.6 [1.6, 1.9]	1.6 [1.6, 3.2]
RNA plasma detectable % (#)^d^	26.3% (5)	37.5% (3)
AIDS % (#)^e^	50.0% (2)	50.0% (2)
Time since first positive test; mean (SD)^f^	125.5 (101.0)	201.1 (104.9)
Meth use characteristics		
Age of first use; mean (SD)^g^	30.0 (12.0)	29.2 (14.6)
Total days used; mean (SD)	1634.8 (2190.8)	1516.2 (1551.5)
Total quantity used; mean (SD)^h^	1058.7 (1663.4)	1593.4 (2396.4)

Key: ^a^ *n* = 8, ^b^Nadir CD4 count is self-reported, *n* = 16; ^c^in log copies/mL, *n* = 27; _ _^d^<50 cp/mL, *n* = 27; ^e^AIDS status based on the 1993 CDC classification scheme, *n* = 8; ^f^time since first positive test is calculated in months, *n* = 8; ^g^ *n* = 25, ^h^total quantity is in grams. Note: no significant differences were observed for any of the reported variables.

**Table 2:** Participant intervention feedback as provided on a standardized questionnaire: text message ease of understanding/problems, satisfaction, self-perceived efficacy, and future direction.

Question	iTAB (*n* = 17)	Control (*n* = 9)
*Text message ease of understanding/problems *		
I had difficulties understanding the text messages		
Not at all	16 (94%)	8 (89%)
A little bit	0 (0%)	1 (11%)
Moderately	1 (6%)	0 (0%)
Quite a bit	0 (0%)	0 (0%)
Very much	0 (0%)	0 (0%)
Receiving text messages interfered with my daily activities		
Not at all	13 (76%)	9 (100%)
A little bit	1 (6%)	0 (0%)
Moderately	2 (12%)	0 (0%)
Quite a bit	0 (0%)	0 (0%)
Very much	1 (6%)	0 (0%)
*Satisfaction *		
How would you rate your overall satisfaction of participating in this study?		
Extremely unsatisfied	0 (0%)	1 (11%)
Somewhat unsatisfied	0 (0%)	0 (0%)
Neither unsatisfied nor satisfied	1 (6%)	2 (22%)
Somewhat satisfied	5 (29%)	2 (22%)
Extremely satisfied	11 (65%)	4 (44%)
*Self-perceived efficacy *		
Do you feel that the daily text message, “Have you done anything in the past 24 hours?” made you use methamphetamine		
A lot less	6 (35%)	2 (22%)
A little less	6 (35%)	3 (33%)
About the same	4 (24%)	4 (44%)
A little more	0 (0%)	0 (0%)
A lot more	1 (6%)	0 (0%)
The intervention made my overall ART medication adherence		
Much worse	0 (0%)	1 (11%)
A little worse	2 (12%)	0 (0%)
About the same	5 (29%)	3 (33%)
A little better	4 (24%)	5 (56%)
Much better	6 (35%)	0 (0%)
*Future direction *		
I would participate in similar studies in the future		
Not at all	0 (0%)	1 (11%)
A little bit	1 (6%)	0 (0%)
Moderately	3 (18%)	0 (0%)
Quite a bit	1 (6%)	1 (11%)
Very much	12 (71%)	7 (78%)
A text messaging intervention could be helpful to me in the future		
Not at all	1 (6%)	1 (11%)
A little bit	1 (6%)	0 (0%)
Moderately	3 (18%)	3 (33%)
Quite a bit	2 (12%)	1 (11%)
Very much	10 (59%)	4 (44%)

Note: no significant differences were observed for any of the reported variables.
